# Supplementary material for: Participation bias in the UK Biobank distorts genetic associations and downstream analyses
Source: Nat Hum Behav. 2023 Apr 27;7(7):1216–27. doi: 10.1038/s41562-023-01579-9 (PMC10365993; doi:10.1038/s41562-023-01579-9)
Supplement: Supplementary file 1 — Supplementary Sections 1–5 and Figs. 1–9. [file 41562_2023_1579_MOESM1_ESM.pdf]

nature human behaviour

Article https://doi.org/10.1038/s41562-023-01579-9

Participation bias in the UK Biobank distorts genetic associations and downstream analyses

In the format provided by the authors and unedited

Supplement Material

1. SINTRODUCTION 2
   1.1. A note on target population and representativeness 2

2. SMETHODS 2
   2.1. Coding of variables included from the UKBB and HSE 2
   2.2. Coding of variables harmonized across the UKBB, HSE and the UK Census Microdata 4
   2.3. Genotyping, imputation and quality control in the UK Biobank 4

3. SRESULTS 5
   3.1. Genome-wide association study on the liability to UKBB participation 5
   3.2. Probability weighted genome-wide association analyses on UK Biobank traits 5
   3.3. Weighted SNP heritability and genetic correlation estimates 5
   3.4. Effect of participation bias on Mendelian Randomization estimates 5

4. SFIGURES 6
   sFigure 1. Estimated correlations among harmonized variables in the HSE and the UK Census Microdata 6
   sFigure 2. Weighted and unweighted genome-wide analyses: number of genome-wide variants 7
   sFigure 3. Weighted and unweighted genome-wide analyses: SNP effects 8
   sFigure 4. Autosomal genome-wide association analyses on biological sex 9
   sFigure 5. Genome-wide association study on UKBB participation – Manhattan plot 10
   sFigure 6. Genome-wide association study on UKBB participation – QQ plot 10
   sFigure 7. SNP heritability estimates in weighted (wGWA) and standard genome-wide (GWA) analyses 11
   sFigure 8. Genetic correlation estimates from weighted and standard genome-wide analyses 12
   sFigure 9. Effect of participation bias on exposure-outcome associations obtained from Mendelian Randomization 13

5. SREFERENCES 14

1. sIntroduction

1.1. A note on target population and representativeness

A target population is a broader group of individuals from which a study sample is drawn and to which the study results should generalize to. Depending on the research question, this might be babies born in India in 2007[1], US woman diagnosed with breast cancer[2], or – as is the case for the UK Biobank – middle-aged to older adults living in the United Kingdom. A representative sample is a subset of that target population that accurately reflects the properties of that group. To ensure representativeness of the group of individuals sampled from the target population, each individual must have the same chance of being included in the sample.

2. sMethods

2.1. Coding of variables included from the UKBB and HSE

Variable	UKBB	HSE	Coding	H	G
Frequency of alcohol use (SR)	About how often do you drink alcohol? (ID: 1558)	How often have you had an alcoholic drink of any kind during the last 12 months?	0=never, 1=few times/year, 2=few times/year, 3=monthly, 4=once or twice/week, 5=three or four days/week, daily	X	
Weekly alcohol use (SR)	In an average week, how many beer/cider/champagne/ wine/spirits/other alcohol) would you drink? (ID: 1588, 1578, 1608, 5364, 1568, 1598)		continuous		X
Physical activity (SR)	Number of days/week of vigorous physical activity 10+ minutes (ID:904)		continuous		X
Sex	Sex of participant	Sex of participant	0=Male/1=Female	X	X
Age	Age of participant	Age of participant	Continuous	X	
Years of education (SR)	At what age did you complete your continuous full-time education? (ID: 845) [note: Individuals with a University degree (ID: 6138) were allocated '19 or over']	At what age did you finish your continuous full-time education at school or college?	14 (or under) /15/16/17/18/19 or over	X	X
Smoking status (SR)	Summary if the current/past smoking status of the participant (ID: 20116)	Have you ever smoked a cigarette, a cigar or a pipe? / Do you smoke nowadays?	0=Never/1=previous/2=current	X	X
Vegetable intake (SR)	About how many heaped tablespoons of cooked vegetables would you eat per day? (ID: 1289)		Continuous		X
Fruit intake (SR)	About how many pieces of fresh fruit would you eat per day? (ID: 1309)		Continuous		X
Income (SR)	What is the average total income before tax received by your household? (ID: 738)	What is your household's income before any deductions for income tax, National Insurance, etc?	1=18k, 2=18k-31k, 3=31k-52k, 4=52k-100k,5=>100k	X	
Household size (SR)	Including yourself, how many people are living together in your household? (ID: 705)	Interviewer collects the names of the people in the household	Categorical, with each category indicating the number of individuals living in the household. 7 = 7 individuals or more.	X	
Employment status (SR)	Which of the following describes your current situation? […] (ID: 6142)	Which of these descriptions applies to what you were doing last week? […]	1=unemployed, 2=employed, 3=economically inactive, 4=retired	X	
Height (M)	Measured using a Seca 202 device (ID: 50)	Measured during the face-to-face household interview	Continuous	X	X

Weight (M)	Measured during the initial Assessment Centre visit (ID: 210002)	Measured during the face-to-face household interview	Continuous	X	
BMI (M)	Derived from height and weight measures	Derived from height and weight measures	Continuous	X	X
BMI (categorical) (M)	Derived from height and weight measures	Derived from height and weight measures	1=underweight (BMI<18.5),<br>2=normal weight (18.5 ≤ BMI <25)<br>3=overweight (25 ≤ BMI <30)<br>4=obese (BMI ≥ 30),	X	
Non-cancer diagnoses (number) (SR)	Number of self-reported non-cancer illnesses (ID: 135)		Continuous		X
Risk taking (SR)	Would you describe yourself as someone who takes risks? (ID: 2040)		0=No/1=Yes		X
Loneliness (SR)	Do you often feel lonely? (ID: 2020)		0=No/1=Yes		X
Diabetes (SR)	Has a doctor ever told you that you have diabetes? (ID: 2443)		0=No/1=Yes		X
Depression/Anxiety (SR)	Seen a psychiatrist for nerves, anxiety, tension or depression (ID: 2100)		0=No/1=Yes		X
LDL (M)	LDL cholesterol (ID: 30780)		Continuous		X
SBP (M)	Systolic blood pressure, automated reading (ID: 4080)		Continuous		X
Reaction time (T)	Reaction time (mean time to correctly identify matches)<br>(ID: 20023)		Continuous		X
Insomnia (SR)	Do you have trouble falling asleep at night or do you wake up in the middle of the night? (ID: 1200)		1=Never/rarely, 2=sometimes, 3=usually		X
Cancer (SR)	Has a doctor ever told you that you have had cancer? (ID: 2453)		0=No/1=Yes		X
Coffee intake (SR)	How many cups of coffee do you drink each day? (ID: 1498)		Continuous		X
Urbanisation (D)	Classification derived by combining each participant's home postcode with data generated from the 2001 census from the Office of National Statistics (ID: 20118)	Degree of urbanisation	1=village/hamlet, 2=town/fringe, 3=urban	X	
Overall health (SR)	In general, how would you rate your overall health? (2178)	How is your health in general?	1=poor; 2=fair, 3=good	X	

D=derived using postcode data; H=used for harmonization and included in the model predicting participation probability; G=included as outcome in genome-wide analyses; M=measured using objective devices; SR=self-reported; T=tested using computerized cognitive test.

2.2. Coding of variables harmonized across the UKBB, HSE and the UK Census Microdata

Variable	UKBB	HSE	Census	Coding
Sex	Sex of participant	Sex of participant	Sex of participant	0=Male/1=Female
Age	Age of participant	Age of participant	Age of participant	40-44 / 45-49 / 50-54 / 55-59 / 60-64 / 65-69
Years of education	At what age did you complete your continuous full-time education? (ID: 845) [note: Individuals with a University degree (ID: 6138) were allocated '19 or over']	At what age did you finish your continuous full-time education at school or college?	Level of highest qualifications:<br>14 (or under)=No academic or professional qualifications<br>15=Other (vocational/foreign/outside UK quals)<br>15=Level 1 (0-4 GCSE, O level, or equivalents)<br>16=Level 2 (5+ GCSE, O level, 1 A level, or equivalents)<br>17=Apprenticeship<br>17=Level 3 (2+ A levels, or equivalents)<br>19 (or over)=Level 4+ (degree, postgrad, professional quals)	14 (or under) /15/16/17/18/19 or over
Employment status	Which of the following describes your current situation? […] (ID: 6142)	Which of these descriptions applies to what you were doing last week? […]	Employment Status based on the International Labour Office (ILO) definition.	1=unemployed, 2=employed, 3=economically inactive, 4=retired
Overall health	In general, how would you rate your overall health? (2178)	How is your health in general?	Self-reported health	1=poor; 2=fair, 3=good

2.3. Genotyping, imputation and quality control in the UK Biobank

488,377 UKBB participants were genotyped, using either the Applied Biosystems UK BiLEVE Axiom Array (807,411 markers for 49,950 participants) or the Applied Biosystems UK Biobank Axiom Array (825,927 markers for 438,427 participants). Poor quality samples were identified using the metrics of missing rate and heterozygosity computed using a set of 605,876 high quality autosomal markers that were typed on both arrays. Imputation was performed using IMPUTE4 with the Haplotype Reference Consortium (HRC) UK10K and the 1000 Genomes Phase 3 dataset as the main imputation reference panels. Detailed genotyping, imputation and quality control (QC) procedures have previously been described[3]. Additional quality control filters for genome-wide analyses were applied to select participants (i.e., exclusion of related individuals, exclusion of non-White British ancestry based on principal components, high missing rate and high heterozygosity on autosomes) and genetic variants (Hardy–Weinberg disequilibrium $P>1\times10^{-6}$, minor allele frequency>1% and call rate>90%).

3. sResults

3.1. Genome-wide association study on the liability to UKBB participation

wGWA on UKBB participation was conducted in N_{eff}=102,215 participants. 28 SNPs reached genome-wide significance ($p<5\times10^{-8}$), of which LD-independent 23 SNPs were selected after clumping. sFigure 5 displays the Manhattan plot with positional mapping of genome-wide SNPs associated with the liability to UKBB participation (cf. sTable 6 for annotation and estimates of significant SNPs). The QQ plot is shown in sFigure 6. A lookup of SNP-trait associations estimated in previous GWA analyses showed that UKBB participation-associated variants mostly tapped into age-related outcomes (e.g., cause of death: cancer/dementia/fatty liver disease/pneumonia) (sTable 7).

3.2. Probability weighted genome-wide association analyses on UK Biobank traits

Among all genome-wide hits (1690, with $p<5\times10^{-8}$), overestimation was more common (420 SNPs, 24.85% of all genome-wide SNPs) than underestimation (290 SNPs, 17.16% of SNPs). Change in direction of SNP effects was rare, as was the case for only one of all 1690 identified SNPs (rs2163971 on smoking status). However, the effects were only significant in standard GWA ($\hat{\beta}$=0.011, p=1.13e-09) but not weighted GWA ($\hat{\beta}_w$=-0.001, p_w=0.776).

3.3. Weighted SNP heritability and genetic correlation estimates

A number of the assessed trait-pairs were significantly underestimated or overestimated as a result of participation bias. Change in direction of genetic correlations as a result of participation bias was less present. While a number of genetic correlations showed opposite signs between *rg* and rg_w (17 out of the 153 assessed trait pairs), none of these rg_{DIFF} (*rg*- rg_w) were significantly different (p_{FDR}<0.05). For example, the largest rg_{DIFF} with opposite signs in *rg* and rg_w was present for *rg*(depression/anxiety, vegetable intake) [*rg*=0.19; *p*=4.3e-05 versus rg_w=-0.12; *p*=0.45, FDR-corrected *p*-value for rg_{DIFF} = 1] and *rg*(number of illnesses, vegetable intake) [*rg*=0.19; *p*=7e-07 versus rg_w=-0.01; *p*=0.9, FDR-corrected *p*-value for rg_{DIFF} = 1].

3.4. Effect of participation bias on Mendelian Randomization estimates

Of all exposure-outcome associations tested (k=234), 14 (6%) estimates were either overestimated or underestimated. Significant (p_{FDR}<0.05) differential effects were only present for two of the exposure-outcome associations tested (education on BMI; smoking status on fruit consumption). There was little evidence of bias resulting in changes in direction of MR estimates. The largest difference between $\hat{\alpha}$ and $\hat{\alpha}_w$ resulting from opposite effects was present for fruit intake on LDL cholersterol ($\hat{\alpha}$=0.03; *p*=0.83 versus $\hat{\alpha}_w$=-0.12; *p*=0.47) and smoking status on physical activity ($\hat{\alpha}$=0.07; *p*=0.091 versus $\hat{\alpha}_w$=-0.04; *p*=0.45).

4. sFigures

sFigure 1. Estimated correlations among harmonized variables in the HSE and the UK Census Microdata

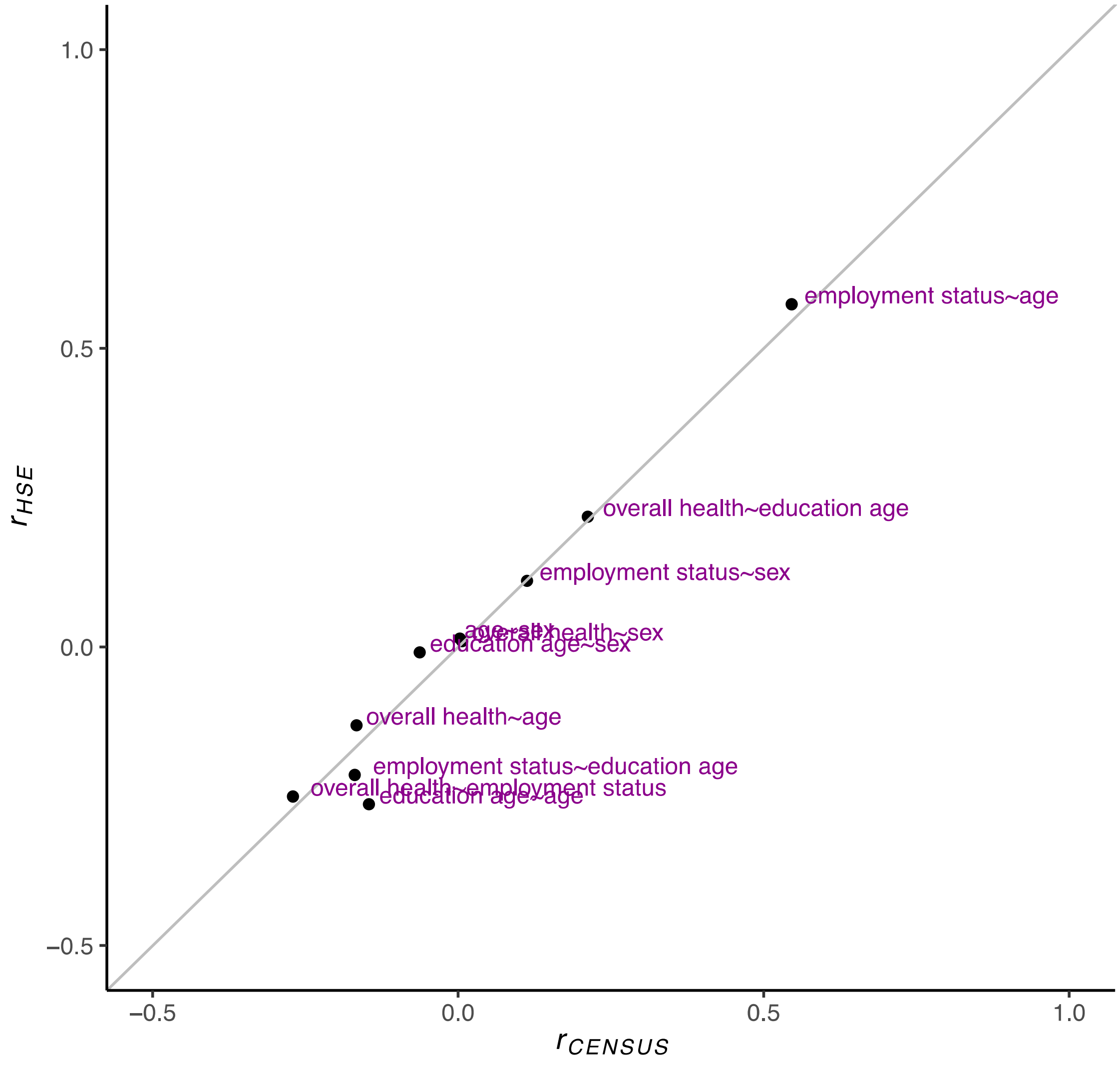

sFigure 2. Weighted and unweighted genome-wide analyses: number of genome-wide variants

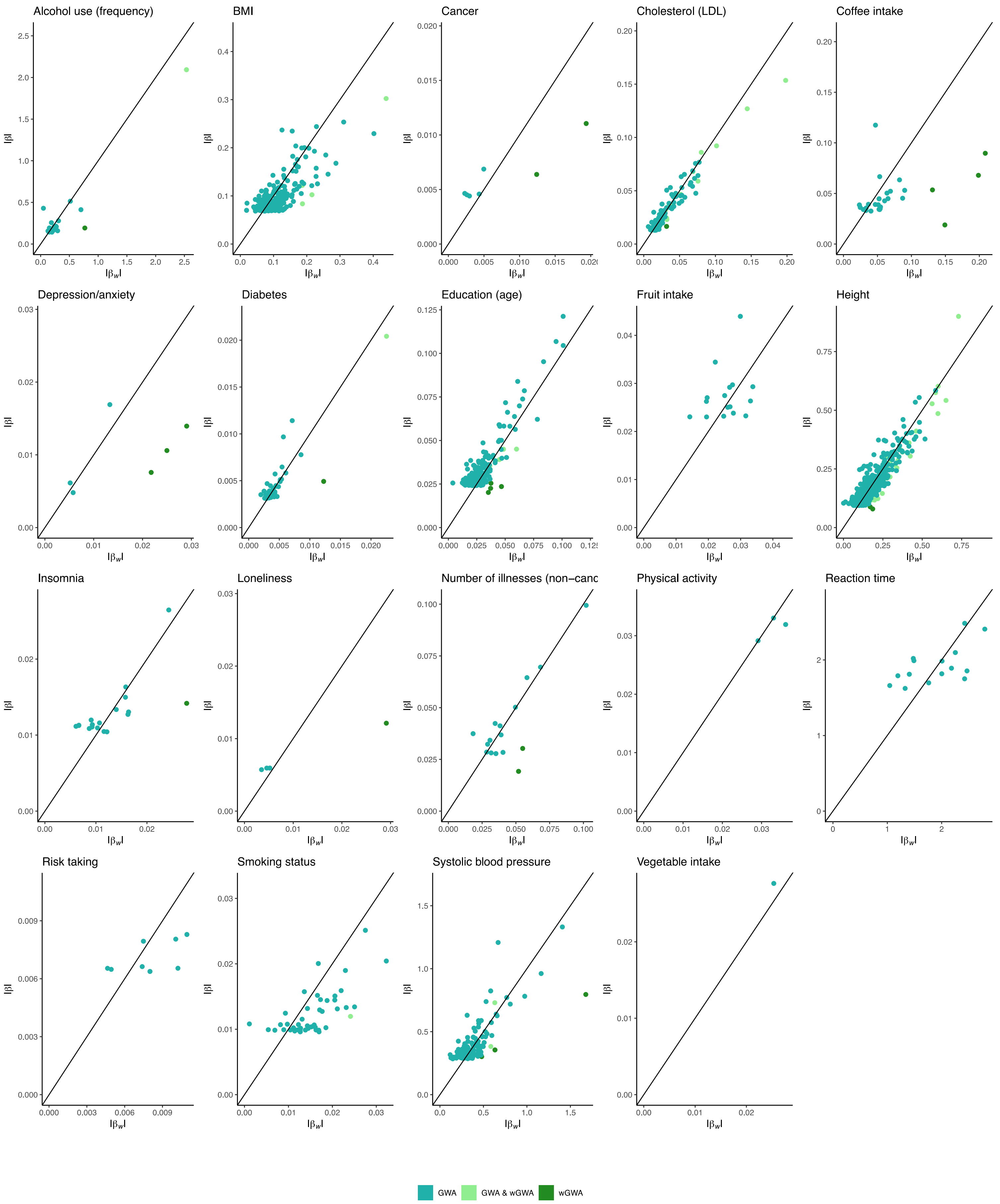

sFigure 3. Weighted and unweighted genome-wide analyses: SNP effects

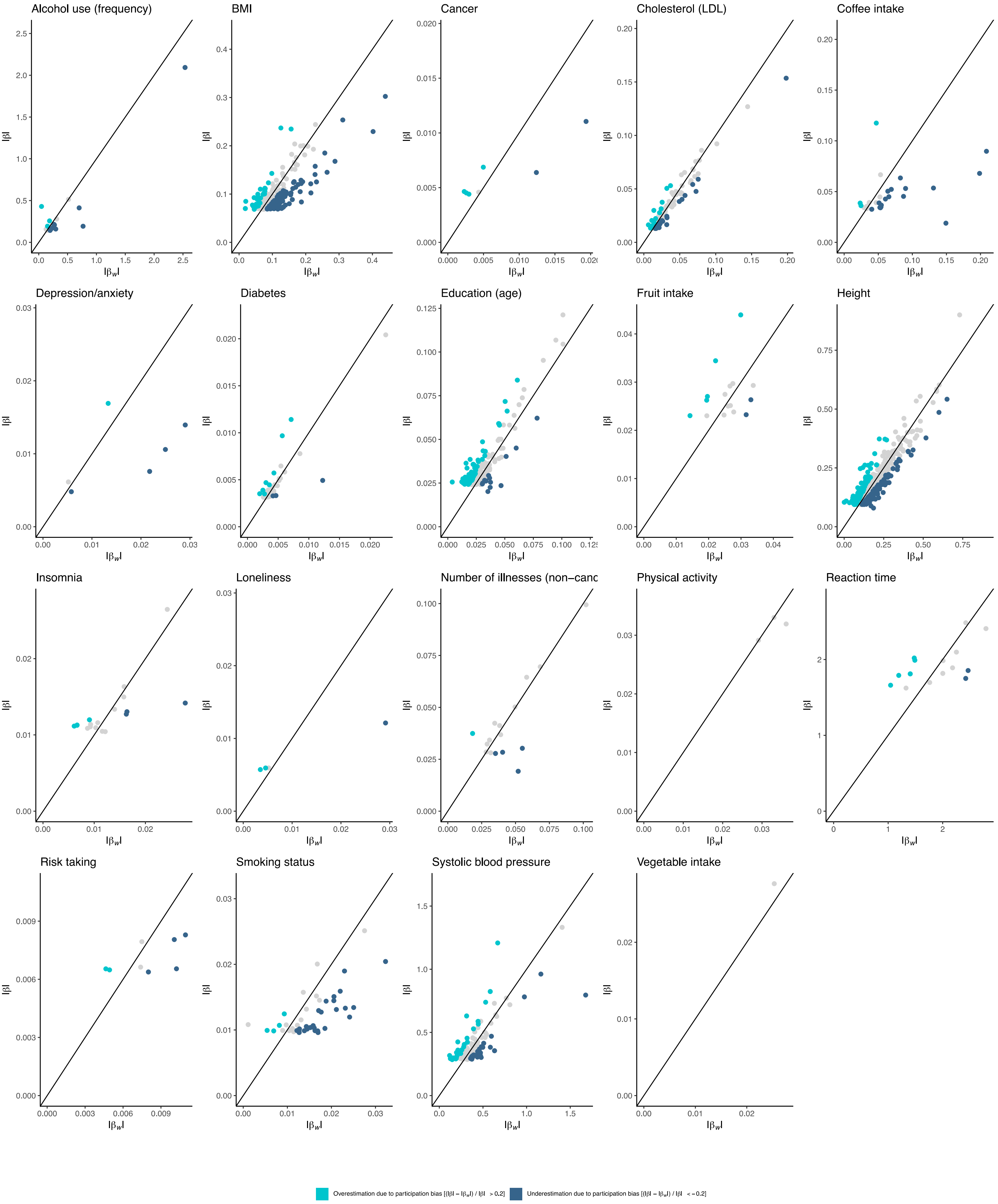

sFigure 4. Autosomal genome-wide association analyses on biological sex

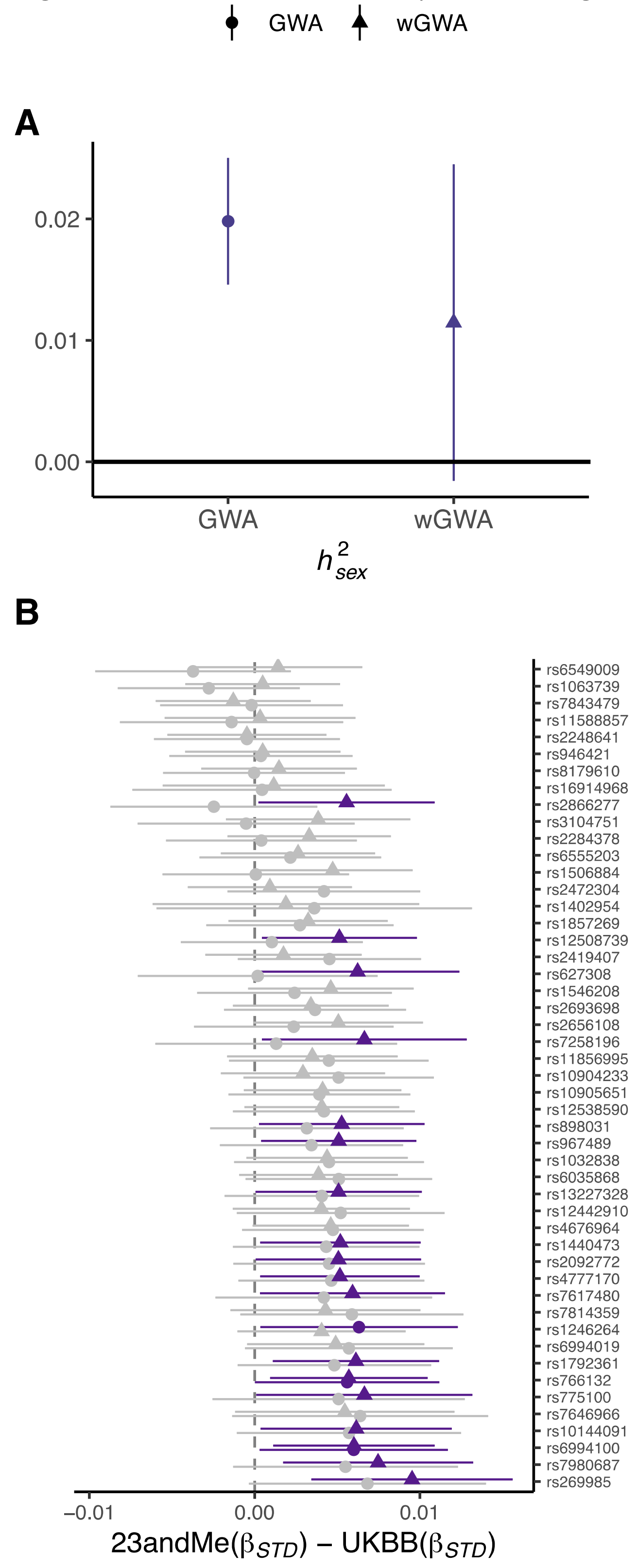

Panel (**A**) displays the SNP heritability (h^2_{sex}) estimates and corresponding 95% confidence intervals of sex-associated variants obtained from standard GWA and wGWA. Panel (**B**) displays the effects of 49 autosomal variants on sex, comparing standardized estimates obtained from standard GWA and wGWA to estimates obtained from an independent sample of >2,400,000 volunteers. The plotted estimates correspond to the

difference between the two β_{STD} (UK Biobank and 23andMe), the error bars reflect the corresponding 95% confidence intervals.

sFigure 5. Genome-wide association study on UKBB participation – Manhattan plot

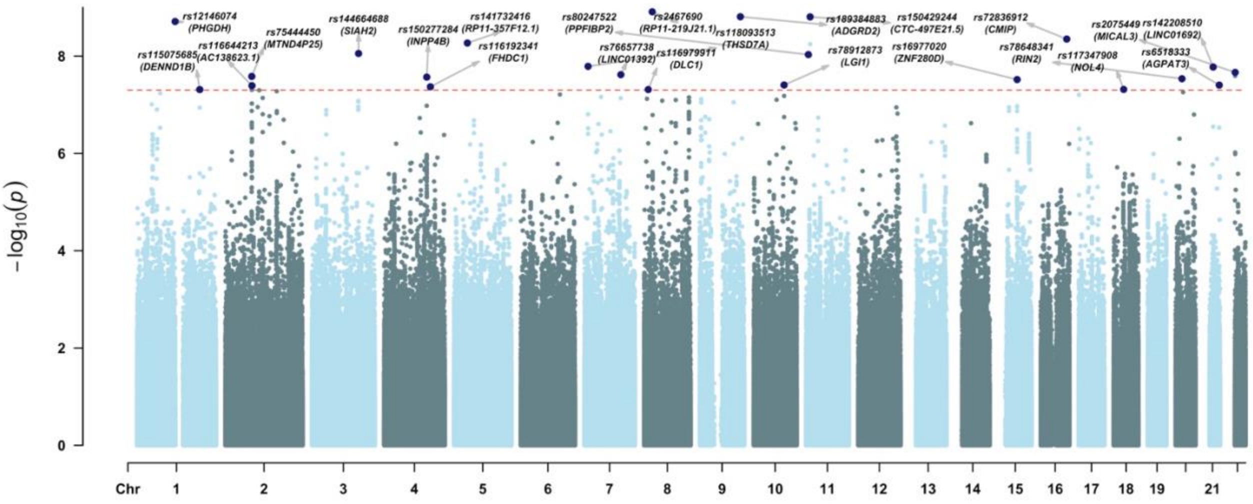

Displayed is the Manhattan plot of the genome-wide association study on the liability to UKBB participation. Labels are provided for the top LD-independent genome-wide significant SNPs (i.e., SNPs above the horizontal line, with $p<5\times10^{-8}$ from two-sided tests) and gene names obtained through positional mapping. The x-axis refers to chromosomal position, the y-axis refers to the *p*-value on a -log10 scale.

sFigure 6. Genome-wide association study on UKBB participation – QQ plot

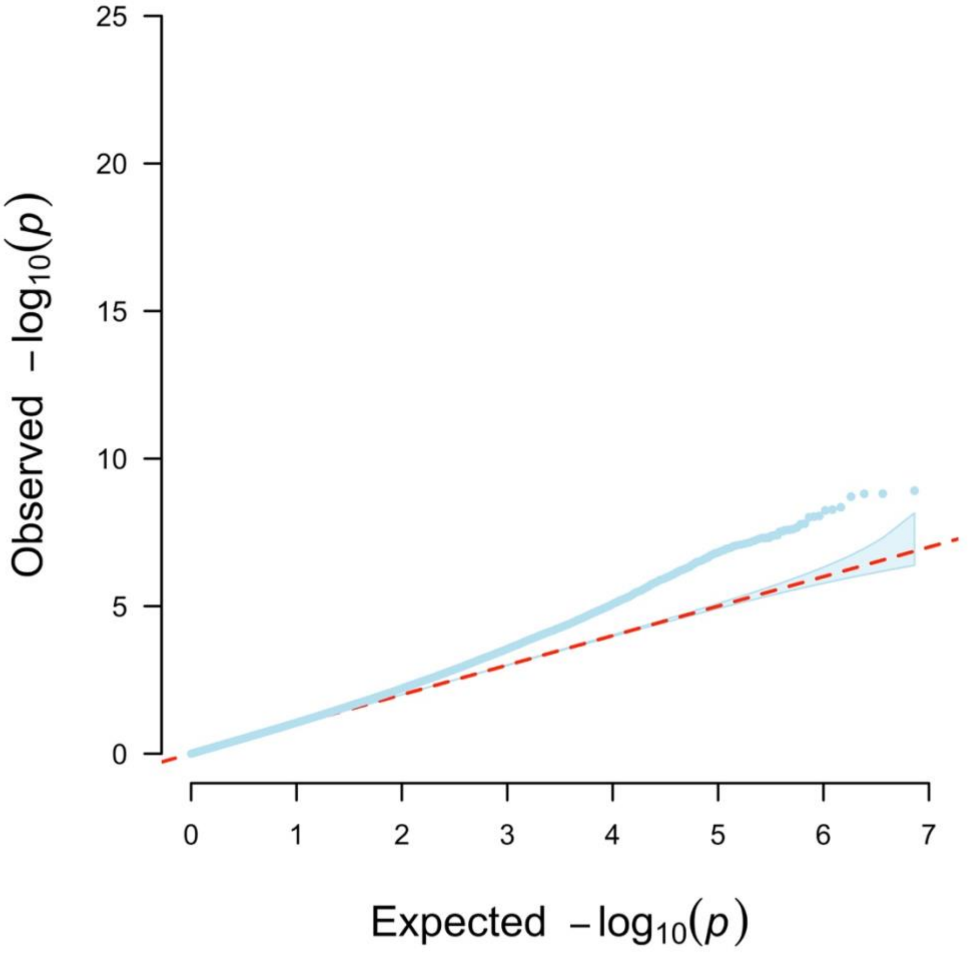

sFigure 7. SNP heritability estimates in weighted (wGWA) and standard genome-wide (GWA) analyses

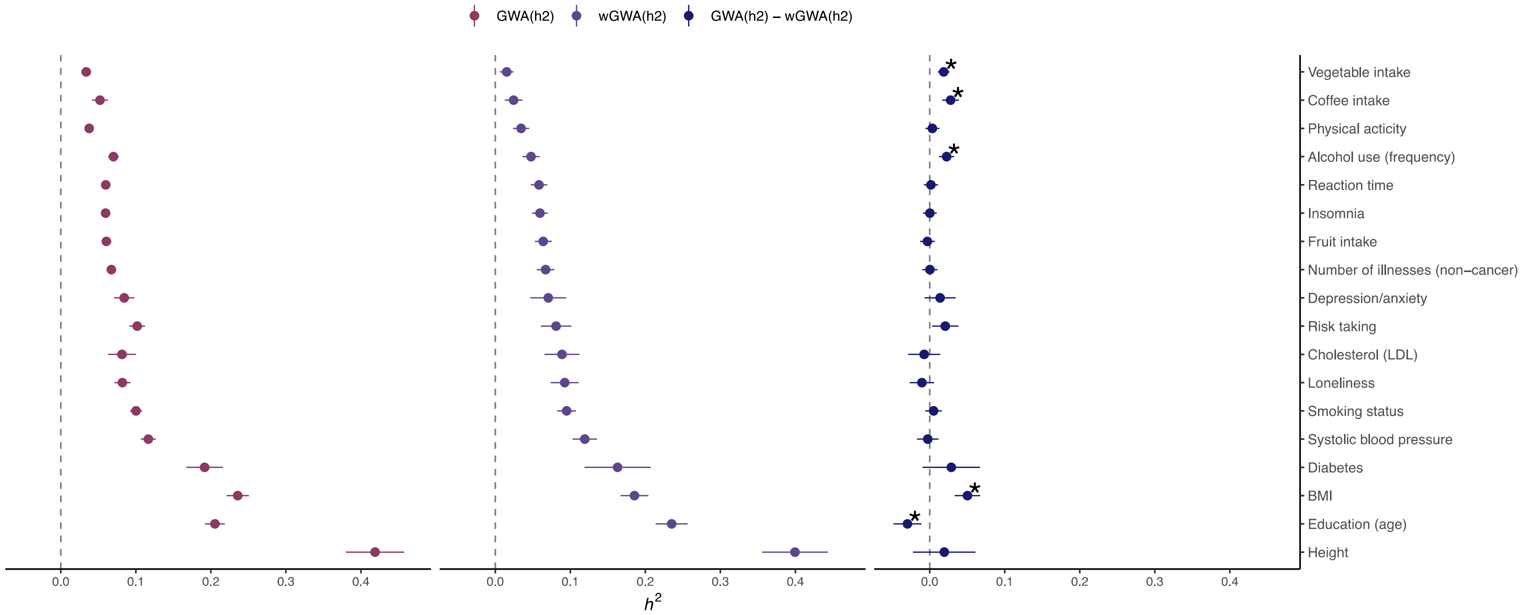

LDSC heritability (h^2) estimates (with corresponding 95% confidence intervals), obtained using the output from standard (unweighted) GWA analyses [GWA(h2)] and probability weighted GWA [wGWA(h2)]. The right panel displays the differences in SNP heritability between standard and weighted GWA (h^2 - h_w^2). (*) Estimates showing significant differences (p_{FDR}<0.05). All p-values are from two-sided tests and corrected for multiple testing using FDR-correction (controlled at 5%).

sFigure 8. Genetic correlation estimates from weighted and standard genome-wide analyses

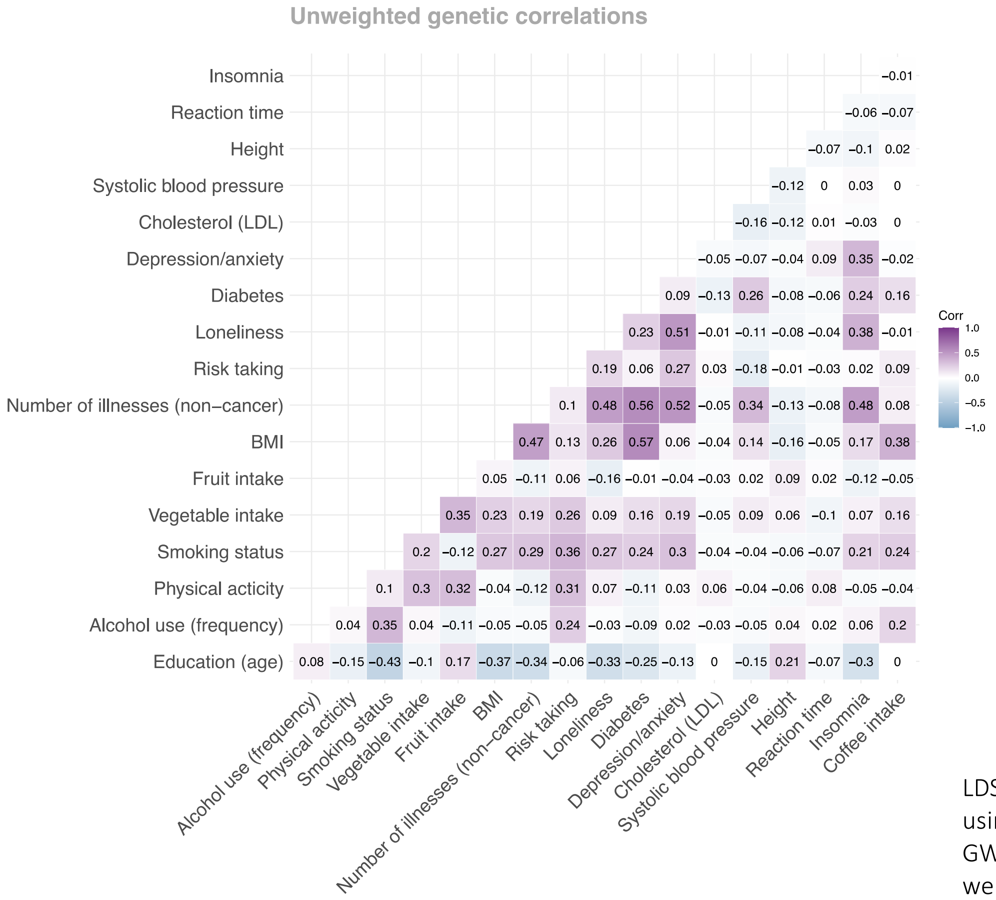

LDSC genetic correlations estimates, obtained using the output from standard (unweighted) GWA analyses (upper panel) and probability weighted GWA (lower panel)

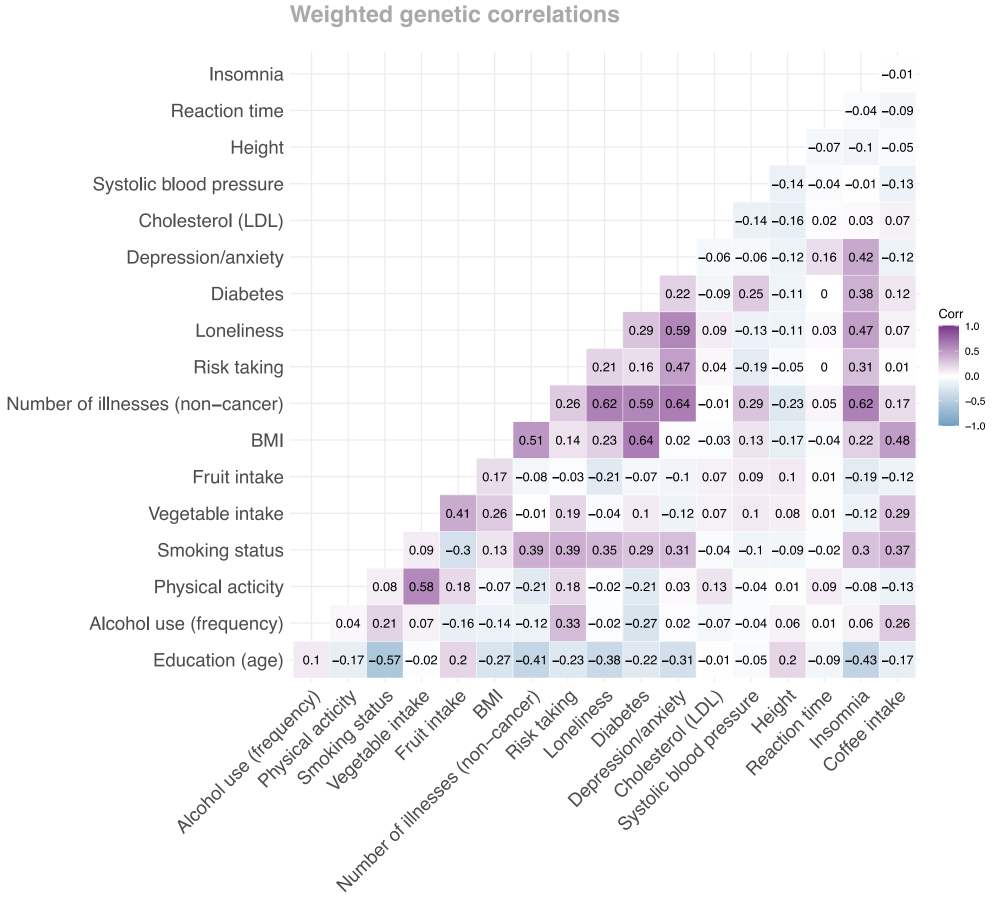

sFigure 9. Effect of participation bias on exposure-outcome associations obtained from Mendelian Randomization

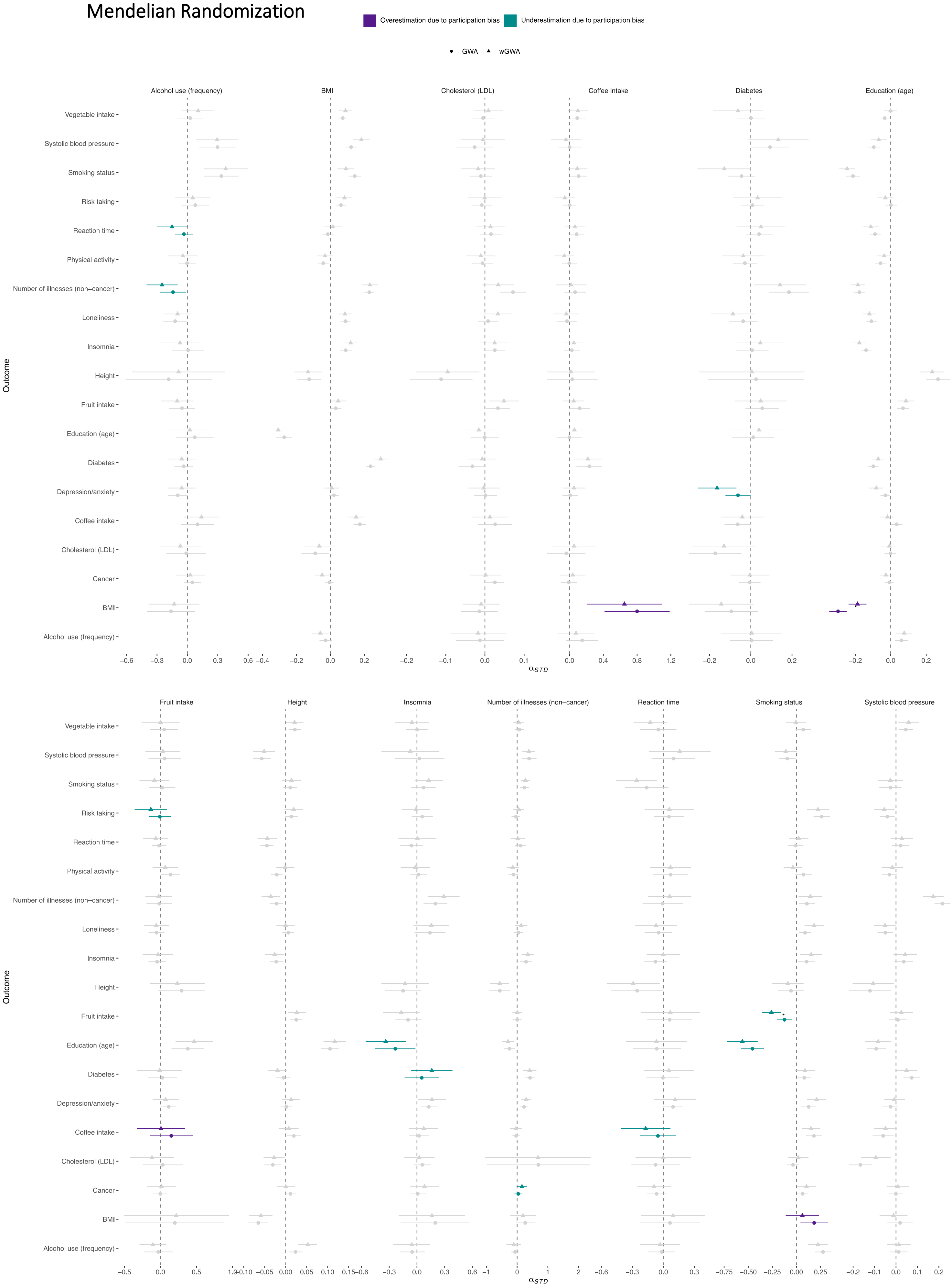

Summary of results of Mendelian Randomization (MR) estimates obtained from weighted ($\hat{\alpha}_w$ ▲) and standard GWA ($\hat{\alpha}$ ●). The asterisks (*) highlight results where $\hat{\alpha}$ and $\hat{\alpha}_w$ showed significant (p_{FDR}<0.05) differences. The error bars signify the 95% confidence intervals corresponding to $\hat{\alpha}$. All p-values are from two-sided tests and corrected for multiple testing using FDR-correction (controlled at 5%).

5. sReferences

1. Singh A, Yadav A, Singh A. Utilization of postnatal care for newborns and its association with neonatal mortality in India: An analytical appraisal. *BMC Pregnancy Childbirth*. 2012;12(1):33. doi:10.1186/1471-2393-12-33
2. Darby SC, McGale P, Taylor CW, Peto R. Long-term mortality from heart disease and lung cancer after radiotherapy for early breast cancer: prospective cohort study of about 300 000 women in US SEER cancer registries. *Lancet Oncol*. 2005;6(8):557-565. doi:https://doi.org/10.1016/S1470-2045(05)70251-5
3. Bycroft C, Freeman C, Petkova D, et al. The UK Biobank resource with deep phenotyping and genomic data. *Nature*. 2018;562(7726):203-209. doi:10.1038/s41586-018-0579-z
